# Augmented insights and minor adjustments to the role of adhesin proteins in *Acinetobacter baumannii* infections

**DOI:** 10.1128/iai.00066-25

**Published:** 2025-05-23

**Authors:** Dongmei Xiao, Huaichang Zhong, Qionghua Huang, Mei Cha

**Affiliations:** 1 Department of Hospital Infection Management, The First People's Hospital of Shuangliu District (West China Airport Hospital of Sichuan University) 12530, Chengdu, China; 2 Department of Public Health, Chengdu Women's and Children's Central Hospital, School of Medicine, University of Electronic Science and Technology of China 12599, Chengdu, China; University of California Merced, Merced, California, USA

**Keywords:** *Acinetobacter baumannii*, infection, adhesin, vaccine

## LETTER

Pereira et al. (1) investigated the role of adhesin proteins in controlling *Acinetobacter baumannii* infections, identifying potential therapeutic targets through an analysis of studies involving bioinformatics, immunization, and antibody-mediated therapies. Pereira et al. (1) provided valuable insights and reference points for the development of new treatments and preventive strategies against multidrug-resistant *A. baumannii* infections, thereby aiding researchers and clinicians in exploring alternative solutions, such as vaccines and immunotherapies. We read their article with great interest and would like to offer some comments in light of our knowledge.

First, Pereira et al. (1) argued that since *A. baumannii* was responsible for nosocomial infections, developing a vaccine against this pathogen may not be feasible. This perspective is likely based on the complex biological characteristics of the pathogen and the limitations of current technology. *Acinetobacter baumannii* is a highly drug-resistant pathogen that can rapidly adapt to environmental changes and develop resistance, which poses significant challenges for vaccine development. Moreover, its diverse strains and complex pathogenic mechanisms further increase the difficulty of developing a vaccine.

In fact, many studies have emphasized that vaccination may be a more effective intervention measure for preventing *Acinetobacter baumannii* infections (2, 3). Vaccination, as a preventive measure, can provide immune protection before the infection of pathogens, thereby reducing the incidence and severity of infections. Vaccination can also decrease the need for antibiotics, which in turn reduces the occurrence of antimicrobial resistance. Therefore, despite the technical challenges, vaccine development remains a worthwhile endeavor.

Second, Pereira et al. (1) pointed out that the study by Jackson-Litteken et al. (4) uncovered the significance of a novel invasin-like adhesin (InvL) in promoting bacterial adhesion and invasion in urinary tract epithelial cells *in vitro*. We carefully read the research report by Jackson-Litteken et al. (4) and found that they mentioned the role of InvL in bacterial adhesion to urinary tract epithelial cells in their study, but did not observe any InvL-mediated internalization of bacteria into the cells. The claim that InvL has invasive functions is not supported by the research findings of Jackson-Litteken et al. (4). Therefore, a more accurate description would be that Jackson-Litteken et al. (4) confirmed the role of InvL in bacterial adhesion, not invasion.

Third, Pereira et al. pointed out that therapies against *A. baumannii* should target adhesin proteins. We believe that this statement requires some supplementation and clarification. Therapeutic strategies against *A. baumannii* can indeed include targeting adhesin proteins, as these proteins play a key role in the bacterial infection process. However, adhesin proteins are just one of several potential therapeutic targets. In addition to adhesin proteins, *A. baumannii* secretes other virulence factors, such as phospholipase C and glycoprotease CpaA (5, 6), which can also serve as therapeutic targets. Therefore, when developing therapeutic strategies against this pathogen, multiple other targets should be considered. This approach could provide a more comprehensive solution to address the complexity and drug resistance of *A. baumannii*.

Finally, we noticed a minor discrepancy in the article by Pereira et al. (1). In the abstract of their article, they stated that they conducted a systematic review of *A. baumannii* adhesin research from the last decade 2013–2023. However, it should be clarified that their review actually covered the period from January 2013 to July 2024.

In conclusion, Pereira et al. (1) have provided valuable insights into the role of adhesin proteins in controlling *Acinetobacter baumannii* infections. In this letter, we have offered additional perspectives and made minor corrections to certain points in their article. We hope that these efforts will help readers gain a better and more comprehensive understanding of the work by Pereira et al. (1).

## EDITOR'S NOTE

**Ed. Note**: The authors of the original article declined to respond.
